# Cultivation of algal biofilm using different lignocellulosic materials as carriers

**DOI:** 10.1186/s13068-017-0799-8

**Published:** 2017-05-04

**Authors:** Qi Zhang, Cuixia Liu, Yubiao Li, Zhigang Yu, Zhihua Chen, Ting Ye, Xun Wang, Zhiquan Hu, Shiming Liu, Bo Xiao, Shiping Jin

**Affiliations:** 1 School of Energy and Power Engineering, Huazhong University of Science and Technology, Wuhan, 430074 China; 2 School of Energy and Environmental Engineering, Zhongyuan University of Technology, Zhengzhou, 450007 China; 3 School of Resources and Environmental Engineering, Wuhan University of Technology, Wuhan, 430070 China; 4 Department of Civil and Environmental Engineering, The Hong Kong University of Science and Technology, Hong Kong, China; 5 School of Environment, Henan Normal University, Xinxiang, 453007 China; 6 School of Environmental Science and Engineering, Huazhong University of Science and Technology, Wuhan, 430074 China

**Keywords:** Lignocellulosic materials, Algal biofilm, Photo-bioreactor, Surface roughness

## Abstract

**Background:**

Algal biofilm technology is recently supposed to be a promising method to produce algal biomass as the feedstock for the production of biofuels. However, the carrier materials currently used to form algal biofilm are either difficult to be obtained at a low price or undurable. Commercialization of the biofilm technology for algal biomass production extremely requires new and inexpensive materials as biofilm carriers with high biomass production performances.

**Results:**

Four types of lignocellulosic materials were investigated to evaluate their performance of acting as carriers for algal cells attachment and the relevant effects on the algal biomass production in this study. The cultivation of algal biofilm was processed in a self-designed flat plate photo-bioreactor. The biofilm production and chemical composition of the harvested biomass were determined. The surface physics properties of the materials were examined through a confocal laser-scanning microscopy. Algal biomass production varied significantly with the variation of the carriers (*P* < 0.05). All the lignocellulosic materials showed better performances in biofilm production than poly methyl methacrylate, and the application of pine sawdust as the carrier could gain the maximum biofilm productivity of 10.92 g m^−2^ day^−1^ after 16-day cultivation. In addition, 20.10–23.20% total lipid, 30.35–36.73% crude proteins, and 20.29–25.93% carbohydrate were achieved from the harvested biomasses. Biomass productivity increased linearly as the increase of surface roughness, and Wenzel’s roughness factor of the tested materials, and surface roughness might significantly affect the biomass production through the size of surface morphology and the area of surface (*P* < 0.05).

**Conclusions:**

The results showed that lignocellulosic materials can be efficient carriers for low-cost cultivation of algal biofilm and the enhancement of biomass productivity.

**Electronic supplementary material:**

The online version of this article (doi:10.1186/s13068-017-0799-8) contains supplementary material, which is available to authorized users.

## Background

Algae that are rich in lipids, proteins, and carbohydrates can be utilized as a feedstock for biofuels production [1]. In recent years, algal-based bioenergy, like biodiesel, bioethanol, and biogas, has been increasingly considered as a promising alternative for fossil fuels to deal with the energy crisis and global climate change [2]. Therefore, researchers have paid an increasing attention to large-scale algal cultivation. Currently, the major types of algal culture systems are open ponds and enclosed photo-bioreactors with dry biomass content of about 0.5 and 2–6 g L^−1^, respectively [3]. Owing to (1) the low dry biomass content, (2) similar density to water and (3) micron-size of cells or clusters [4, 5], the harvesting process of algal biomass from suspended culture is the main challenge for the commercialization of algae-based bioenergy, since it involves high-energy consumption processes such as flotation, filtration, ultrasonic aggregation and centrifugation. In addition, these processes generally account for about 20–30% of the total algal biomass production cost [6]. To lower the cost for harvesting algal biomass from the suspended culture, bio-flocculation that is based on algal-bacterial, algal-fungal, or algal–algal interactions has been developed [7]. It is energy efficient and generally without secondary pollution [7]. However, this technology is currently not widely applied, due to the challenge to control bio-flocculation at scale [7]. Another challenge is that algal-bacterial based bio-flocculation often produces low-value products, due to the composition of certain amount of bacterial in the harvested biomass [8]. Algal-fungal based bio-flocculation has been proven to be reliable and efficient in harvesting planktonic algal cells [9, 10], but the efficient separation of the fungi and algal cells challenges its scale-up process [7]. Algal–algal-based bio-flocculation does not require the separation process of the harvested biomass, and energy consumption is negligible [11]. However, species with the ability of self-flocculation is limited, and thus the application of this technology usually needs to engineer non-flocculating algal strains, which will in turn threaten the environment [11].

Due to easier harvesting and system operation, higher mass transfer rate and dry mass content, less water consumption and even lower capital construction cost, algal biofilm technology has been regarded as a promising approach to produce biomass for algae-based bioenergy [12]. This strategy requires that the algal cells can adhere to and grow on a given surface, prior to developing into biofilm. Thus, the cost of harvesting processes can be potentially reduced, since the biomass can be easily scrapped off from the surface [3]. Currently, a variety of algal biofilm systems have been developed to study the growth of attached algae. It is believed that the performances of biomass production in different algal biofilm systems not only rely on the culture conditions, algal strains, scale of the process, but also are significantly affected by the characteristics of materials which support the attached growth.

Undoubtedly, it is worthy to note that the selected materials for algal cell attachment need to be durable, inexpensive, easy to be obtained, nontoxic to algal cells, etc. It would be better if the materials have potential to enhance the biofilm production. Recently, glass fiber nonwoven and plain printing paper were applied to benefit nutrient supplement and algae cell attachment in a Twin-Layer algal biofilm photo-bioreactor, respectively [13]. In a similar Twin-Layer lab-scale photo-bioreactor, filter paper and cellulose acetate membrane (0.45 μm) were applied as the source layer and biofilm carrier instead of glass fiber nonwoven and plain printing paper [14]. Moreover, optimal performance of attached growth in kinds of algae biofilm systems was measured with cotton duct and cotton rope as biofilm carriers [3, 15]. Application of rare and valuable materials such as stainless steel woven meshes with a particle pass size of 47 μm [16] and membrane with pore size of 5 μm [6] as support materials for algae biofilm cultivation was also demonstrated with excellent production performance.

However, most of the materials currently used for algal cell attachment were either expensive (like cellulose acetate membrane and glass filtration fabric) or with high construction cost (like metal meshes with pore size of 47 μm). Additionally, most of those materials were disposable and not durable, and thus were unsuitable for full-scale algal biofilm cultivation. Therefore, this paper made the first attempt to develop a new biofilm technology using lignocellulosic materials as biofilm carriers such as pine sawdust, rice husk, sugar bagasse and oak sawdust, all of which are environmentally friendly, cheap, and renewable with a wide range of distribution around the world [17]. These lignocellulosic materials with large numbers of production every year can act as natural materials for algal cells attachment and the formation of algal biofilm in the biofilm systems. After cultivation, algal biomass can be harvested together with the lignocellulosic materials through scrapping. Amazingly, the harvested blend (mixture of the lignocellulosic materials and algal biomass) can be directly utilized as a feedstock for bioenergy conversion.

Hence, the present study aimed at the cultivation of algal biofilm using the lignocellulosic materials as carriers in a self-designed flat plate photo-bioreactor. The objectives of this study were to: (1) determine the performance of this new technology and the optimal materials for algal attachment, (2) estimate the chemical composition of the harvested algal biomass, (3) reveal the impacts of material surface properties on the algal productivity, and (4) specify the factors affecting the algal growth.

## Methods

### Microalgae strains and lignocellulosic materials

Three algae (*Scenedesmus obliquus* FACHB-416, *Chlorella vulgaris* FACHB-32, and *Oscillatoria tenuis* FACHB-1052) involved in this study were purchased from the Institute of Hydrobiology, Chinese Academy of Science, PR China. BG 11 medium [12] with an initial pH of 6.8 was used as the standard culture medium. All species were grown in 500 mL sterilized BG 11 medium under a light intensity of 120 μmol m^−2^ s^−1^ and temperature of 25 ± 2 °C in a 14/10 h light/dark cycle, and aerated with 2% CO_2_. When the optical density (OD_685_) reached about 0.8–1.0 after 4–7 days’ cultivation, the culture was used as the seed for the subsequent experiments.

The lignocellulosic materials, including pine sawdust (PW), rice husk (RH), oak sawdust (OW) and sugarcane bagasse (SB) were involved in this research. PW was obtained from a furniture factory in Wuhan city, Hubei province, China. RH and OW were collected form a village in Suizhou city, Hubei province, China. SB was gathered from a sugar refinery in Guiping city, Guangxi province, China. Materials were dried under the sun for 15 days. Then the bulk density of the selected sample was tested by a densitometer (HYL-103, Hylology, China). The size distribution and bulk density of the selected materials for biofilm carriers are listed in Additional file 1: Table S1).

### Algal biofilm photo-bioreactor

**Fig. 1:**
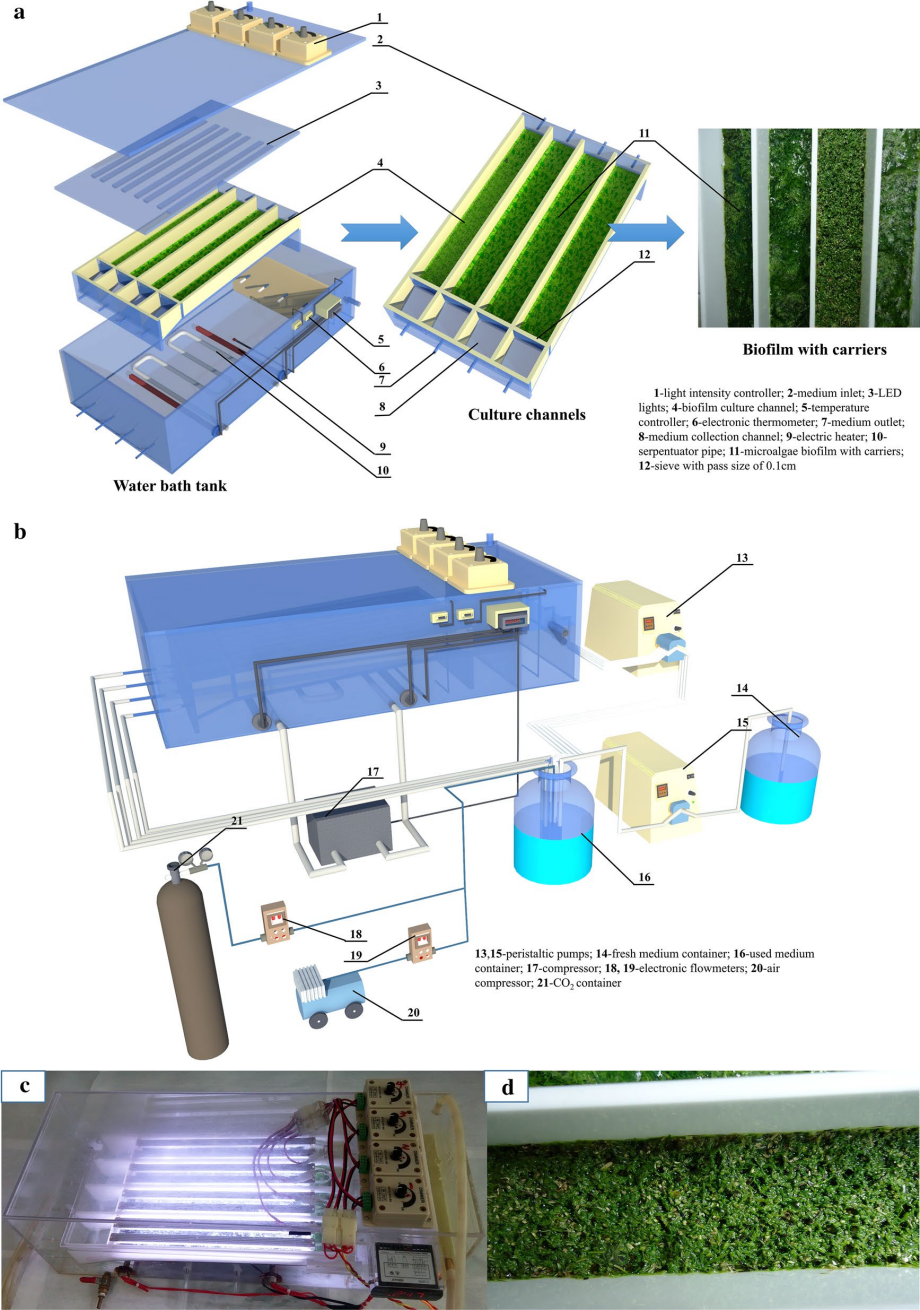
Setup of a lab-scale FPBR system. **a** The schematic diagram of the flat plate algal biofilm photo-bioreactor. **b** The schematic diagram of the whole culture system. **c** The picture of the flat plate algal biofilm photo-bioreactor. **d** The picture of the biofilm with pine sawdust as carriers after 16-day cultivation

A flat plate algal biofilm photo-bioreactor (FPBR) which was coupled with a medium recirculation system and a gas supplement system was constructed (Fig. 1). Figure 1a and c show the setup of the bench-scale FPBR. Figure 1b shows the setup of the whole culture system. The FPBR system was consisted of an inner vessel and an outer case.

**Fig. 2:**
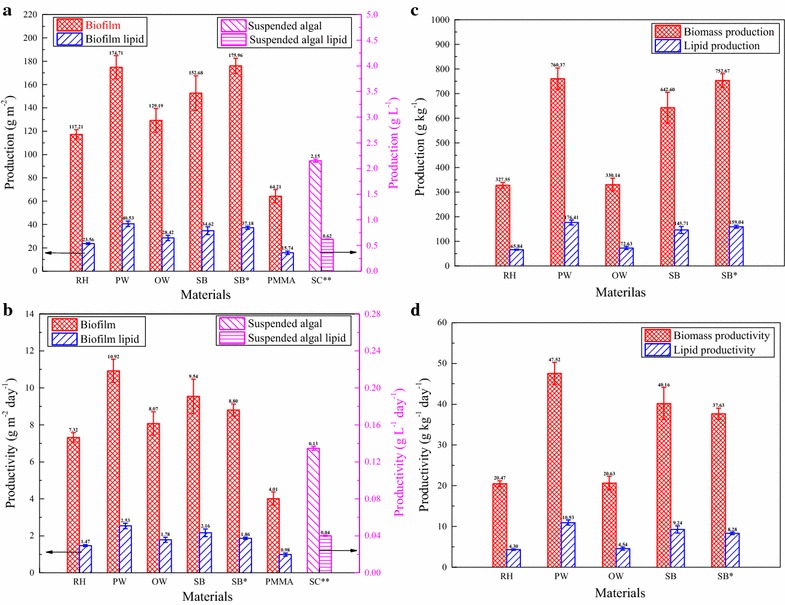
The performance of the biofilm with different materials as carriers and the suspended culture as for biomass production (mean ± SD). **a** Algal biomass and total lipid production calculated as dry weight per growth surface area. **b** Algal biomass and total lipid productivity calculated as dry weight per growth surface area per unit of time. **c** Algal biomass and total lipid production calculated as dry weight per carrier dry weight. **d** Algal biomass and total lipid productivity calculated as dry weight per carrier dry weight per unit of time. *Biofilm growth for 20 days, **suspended culture

**Fig. 3:**
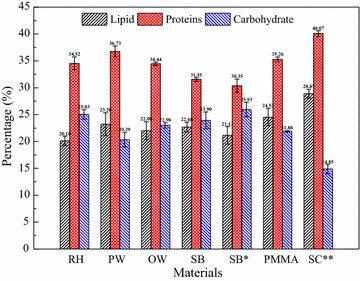
The total lipid, crude proteins, and carbohydrate contents of the harvest algal biomass from the FPBR with different materials as biofilm carriers and the suspended culture (mean ± SD). *Biofilm growth for 20 days, **suspended culture

**Fig. 4:**
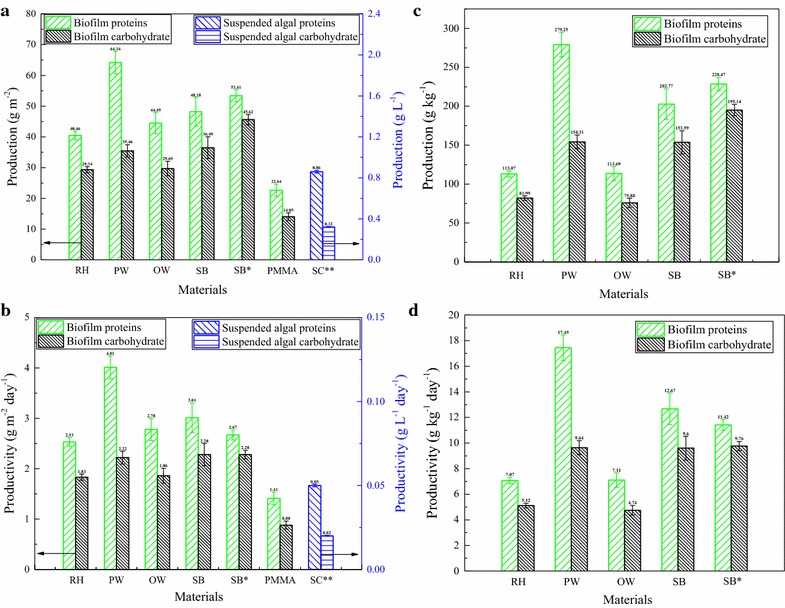
Crude proteins and carbohydrate production of the biofilm with different materials as carriers and the suspended culture (mean ± SD). **a** Crude proteins and carbohydrate production calculated as dry weight per growth surface area. **b** Crude proteins and carbohydrate productivity calculated as dry weight per growth surface area per unit of time. **c** Crude proteins and carbohydrate production calculated as dry weight per carrier dry weight. **d** Crude proteins and carbohydrate productivity calculated as dry weight per carrier dry weight per unit of time. *Biofilm growth for 20 days, **suspended culture

**Fig. 5:**
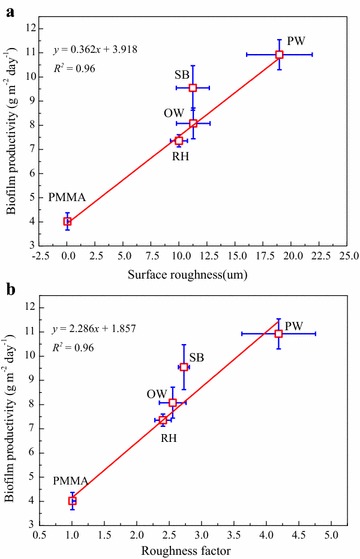
Effect of materials surface physics properties on the algal biofilm productivity (mean ± SD). **a** The relationship between algal biofilm productivity and the surface roughness. **b** The relationship between algal biofilm productivity and the roughness factor

**Fig. 6:**
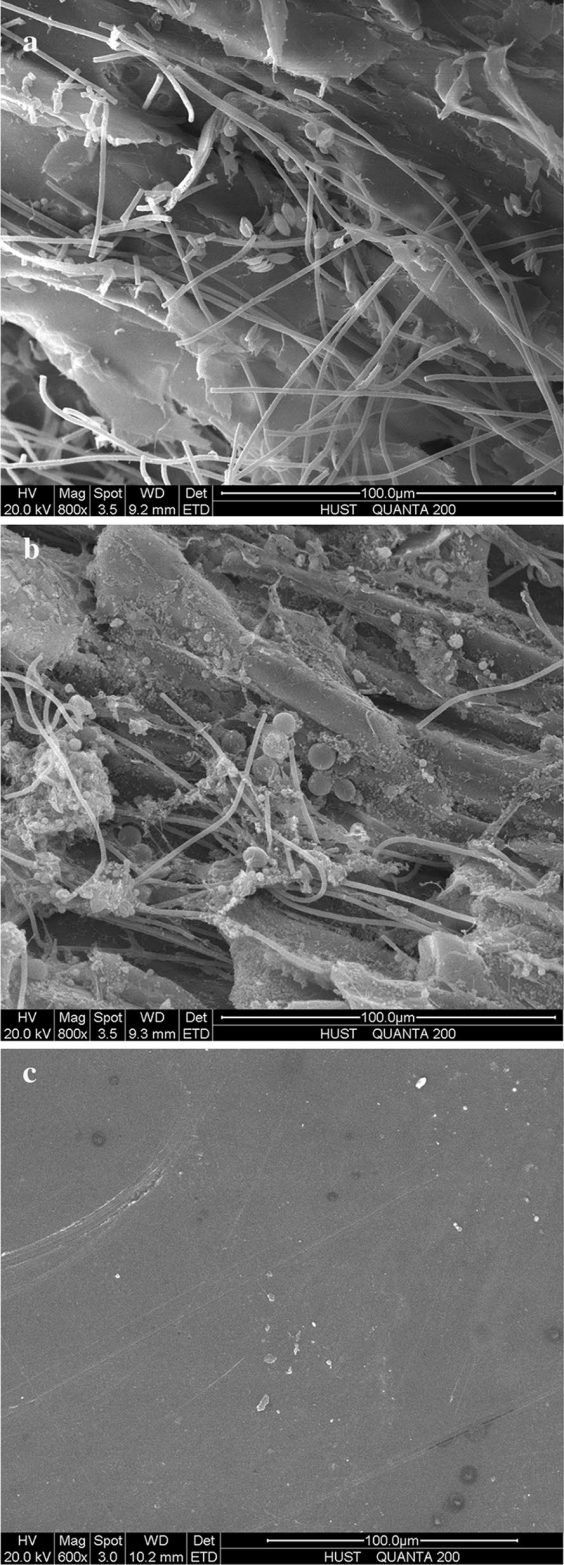
ESEM images of the 1-day-old biofilms with different materials as carriers. **a** Biofilm with PW as carriers. **b** Biofilm with SB as carriers. **c** Biofilm with PMMA as carriers

The outer case made of poly methyl methacrylate (PMMA) was a water bath with 65 cm length, 25 cm width and 20 cm depth. The water bath with 15 L deionized water was used to keep the algal biofilm culture at 25 ± 2 °C. A copper serpentuator pipe (Fig. 1a-10) was set inside the water bath and coupled with a compressor (Fig. 1b-17), and when the temperature was beyond 25.5 °C, the compressor would be started by the temperature controller (EK-3010, Elitech, china) (Fig. 1a-5) to lower the temperature. Moreover, two 100 W electric heaters (Fig. 1a-9) were also fixed inside the water bath and would be powered on by the temperature controller to enhance the temperature when the value was lower than 24.5 °C. The compressor and the electric heaters would not be powered on since the temperature was in the range of 24.5–25.5 °C. In addition, two electronic thermometers (ST-1A, Elitech, china) (Fig. 1a-6) were continuously used to monitor the temperature of the water bath.

Four biofilm culture channels (Fig. 1a-4) and a cover plate with eight LED tubes (Fig. 1a-3) together constituted the inner vessel of the FPBR system. The cover plate was used to enclose the culture channels. Four independent culture channels were partially immerged inside the water bath and kept at 25 ± 2 °C. Each channel (Fig. 1a-4) was 30 cm length, 5 cm width, and 5 cm depth with a biofilm culture area of 150 cm^2^ and a tilt angle of 15°, and illuminated with two LED tubes. The light intensity applied to each channel was accurately controlled with a range of 0–300 μmol m^−2^ s^−1^ by a regulator (JCH-M-DIMMER-8A, China) (Fig. 1a-1) fixed on the outside cover plate. The outside cover plate was utilized to enclose FPBR in order to avoid evaporation and pollution as well. The walls of the channels were identically made of opaque PMMA to avoid unwanted illumination. The lignocellulosic carriers for algal biofilm cultivation was spread out into each channel evenly to generate a thinner layer and just cover the whole culture channel surface (area of 150 cm^2^), while algal strains were inoculated onside the carriers and then developed into biofilm. In addition, there were a PMMA mesh with pore size of 0.1 cm (Fig. 1a-12) and a medium collection channel (Fig. 1a-8) behind the mesh installed at the end of each channel. The PMMA mesh was established in order to avoid any particles of the tested materials being washed away during the higher flow velocity. The medium collection channel with a tilt angle of 30° was set to lead the medium to flow away smoothly so that no medium would be accumulated at the end of the biofilm. Such structure of the reactor could keep the biofilm not be submerged, and thus higher efficiency of mass transfer in gas phase was guaranteed.

The medium recirculation system contained a 15 L used-medium-container (Fig. 1b-16), a 15 L fresh-medium-container (Fig. 1b-14) and two peristaltic pumps (BT100-1F, LONGER, China) (Fig. 1b-13, 15). The gas supplement system consisted of a CO_2_ container (Fig. 1b-21), an air compressor (Fig. 1b-20) and two electronic flowmeters (MF5706, siargo, USA) (Fig. 1b-18, 19). Culture medium was pumped inside each culture channel from the used-medium-container (Fig. 1b-16) at a flow rate of 40 ± 5 mL min^−1^ by a four-channel peristaltic pump (Fig. 1b-13). After crossing the biofilm culturing section, the medium flowed into the four medium collection channels (Fig. 1a-8) and was recirculated into the used-medium-container (Fig. 1b-16). Fresh medium was pumped into the used medium container from a fresh-medium-container (Fig. 1b-14) with a flow rate of 3 mL min^−1^ and about 4.3 L medium was discarded from the used-medium-container (Fig. 1b-16) everyday as well. The used-medium-container (Fig. 1b-16) was aerated with 2% CO_2_ at a flow rate of 4 L min^−1^. Such CO_2_ was performed through the use of two electronic flowmeters to control the relative flow rates of compressed air and CO_2_.

After each batch culture, the whole system was cleaned, and then the pipes and containers were all autoclaved (121 °C, 30 min). 10 g L^−1^ NaClO was used to wipe the surface of PMMA made parts twice for disinfection. Afterwards, the sterilized cold water was applied to clean up the residual NaClO.

### Algal biofilm cultivation

Each lignocellulosic material with a given dosage was spread out into the culture channel to cover the whole culture surface (area of 0.015 m^2^). Then, each channel was inoculated with 30 mL mixed algal culture during the logarithmic phase (10 mL of each algal species). Afterwards, 15 mL BG 11 medium was added every 8 h to keep the substance materials wet. 24 h later, 10 L sterilized BG 11 medium was added into the used-medium-container, and the system began to pump medium continuously from the used-medium-container to the culture channels.

Additional file 1: Table S2 shows the detailed information of the experimental setup and biofilm culture conditions. Experiments 1 and 2 were set to investigate the performance of the tested materials as the carriers for algal cell attachment and the stability together with reproducibility of this algal biofilm culture system. Experiment 3 was set to compare the productivity of PMMA as a biofilm carrier with lignocellulosic biomasses. During this experiment, the channel itself which was made of PMMA plate acted as PMMA carrier for algal biofilm growth. After 16 or 20 days’ cultivation, medium inside the used-medium-container was replaced by 10 L deionized water and the system was processed for another 3 h to wash out the salinity of the biofilm. Finally, the biofilm harvested by scrapping was dried at 105 °C for 48 h for further analysis. Experiment 4 was set to study the liquid-holding capacity of the lignocellulosic materials. System was run with the same condition as the Experiments 1 and 2 with an exception for the inoculation of algal on the material layers. After absorbing water for 1 day, materials were collected and weighed immediately to obtain the saturated moisture contents of the lignocellulosic materials.

### Suspended algal culture

Suspended algal cultivation was conducted in 2 L flasks to compare the performance of algae growth in the biofilm system. The flask was set with 1 L sterilized BG 11 medium and 12 mL algal inoculum (4 mL of each algal species). The growth condition was the same as described in “Microalgae strains and lignocellulosic materials.” Every sixth day, the algal cells harvested through centrifugation in each flask were added into the same flask together with a volume of 1 L fresh sterilized BG 11 medium. The water evaporation lost was balanced daily with sterilized water. After 16 days, suspended algal biomass was harvested through centrifugation, and freeze-dried for further analysis.

### Growth analysis

In this study, the tested materials were firstly oven dried at 105 °C for 24 h before added into the culture channels. After 16 or 20 days’ cultivation, biofilm blend was harvested and oven dried at 105 °C for 24 h. Algal biofilm production (g m^−2^) and productivity (g m^−2^ day^−1^) were calculated as follows:

1 $${\text{Biomass production}} = {{\left[ {W_{\rm b} - \left( {1 - \alpha } \right)W_{\rm t} } \right]} \mathord{\left/ {\vphantom {{\left[ {W_{\rm b} - \left( {1 - \alpha } \right)W_{\rm t} } \right]} {0.015}}} \right. \kern-0pt} {0.015}}$$

2 $${\text{Biomass productivity}} = {{\left[ {W_{\rm b} - \left( {1 - \alpha } \right)W_{\rm t} } \right]} \mathord{\left/ {\vphantom {{\left[ {W_{\rm b} - \left( {1 - \alpha } \right)W_{\rm t} } \right]} {0.015t}}} \right. \kern-0pt} {0.015t}},$$

where $W_{\rm b}$ is the weight of the harvested and oven dried biofilm blend, the mixture of algal biomass, and lignocellulosic carriers; $W_{\rm t}$ is the weight of the tested carriers before cultivation ($W_{\rm t} = 0$ for PMMA); $\alpha$ is the material mass loss rate (1–6.5%) which listed in Additional file 1: Table S1, preliminary study found that there had a slightly mass loss of the tested carriers due to the washing away of the micron-scale particles and the solubilization of light organic compounds; 0.015 is the plan view surface area of a single channel; and $t$ is the culture period. For the suspended culture, the biomass production (g L^−1^) and productivity (g L^−1^ day^−1^) were calculated followed the Eqs. (3) and (4):

3 $${\text{Suspended algal production}} = {{W_{\rm s} } \mathord{\left/ {\vphantom {{W_{\rm s} } V}} \right. \kern-0pt} V}.$$

4 $${\text{Suspended algal productivity}} = {{W_{\rm s} } \mathord{\left/ {\vphantom {{W_{\rm s} } {\left( {V * t} \right)}}} \right. \kern-0pt} {\left( {V * t} \right)}},$$

where $W_{\rm s}$ is the weight of the harvested and freeze-dried suspended algal biomass, $V$ is the volume of the culture medium. In addition, the potential for algal biomass production offered by a unit weight of materials through this new technology was determined. The biomass production and productivity was represented by g kg^−1^ and g kg^−1^ day^−1^, respectively, as follows:

5 $${\text{Biomass production}} = {{\left[ {W_{\rm b}^{{}} - \left( {1 - \alpha } \right)W_{\rm t} } \right]} \mathord{\left/ {\vphantom {{\left[ {W_{\rm b}^{{}} - \left( {1 - \alpha } \right)W_{\rm t} } \right]} {0.001W_{\rm t} }}} \right. \kern-0pt} {0.001W_{\rm t} }}$$

6 $${\text{Biomass productivity}} = {{\left[ {W_{\rm b}^{{}} - \left( {1 - \alpha } \right)W_{\rm t} } \right]} \mathord{\left/ {\vphantom {{\left[ {W_{\rm b}^{{}} - \left( {1 - \alpha } \right)W_{\rm t} } \right]} {\left( {0.001t * W_{\rm t} } \right)}}} \right. \kern-0pt} {\left( {0.001t * W_{\rm t} } \right)}}$$

The saturated moisture contents (%) of the lignocellulosic materials were calculated using Eq. (7):

7 $${\text{Saturated moisture content}} = \left[ {{{\left( {W_{\rm t}^{'} - W_{\rm t} } \right)} \mathord{\left/ {\vphantom {{\left( {W_{\rm t}^{'} - W_{\rm t} } \right)} {W_{\rm t}^{'} }}} \right. \kern-0pt} {W_{\rm t}^{'} }}} \right] \times 100\%,$$

where $W_{\rm t}^{'}$ is the weight of the harvested material from Experiment 4. In addition, the liquid-holding capacity (g/g) of the lignocellulosic materials (the mass of medium held by unit weight of tested material) was defined as follows:

8 $${\text{Liquid-holding capacity}} = {{\left( {W_{\rm t}^{'} - W_{\rm t} } \right)} \mathord{\left/ {\vphantom {{\left( {W_{\rm t}^{'} - W_{\rm t} } \right)} {W_{\rm t} }}} \right. \kern-0pt} {W_{\rm t} }}$$

### Environmental scanning electron microscopy (ESEM)

Environmental scanning electron microscopy images of the 1-day-old biofilm with PW, SB and PMMA as carriers were obtained by an environmental scanning electron microscope (QUANTA 200, FEI, Holland). First, samples were fixed with 2.5% glutaraldehyde for 4 h and then gently washed with phosphate buffer solution (137 mM NaCl, 2.7 mM KCl, 10 mM Na_2_HPO_4_, 2 mM KH_2_PO_4_, pH 7.4) for three times. Second, 30, 50, 70, 85, and 100% (v/v) ethanol was used to sequentially dehydrate the fixed samples for 15 min. Third, ethanol inside the dehydrated samples was exchanged by isoamyl acetate within 20 min for twice. After these processes, samples were frozen at −20, −40, −80 °C for 4 h, respectively, and then freeze-dried for 12 h. Finally, the dried samples were sputter coated with a thin gold layer for the observation of surface morphology.

### Chemical composition of the harvested biomass

Biofilm blend was first stirred with 200 mL sterilized BG 11 medium at 2000 rpm/min for 10 min and then sonicated in an ultrasonication bath (KQ-100DB, KUNSHAN, China) for 40 min, and thus cells were resuspended. Suspended biomass was harvested through centrifugation, then freeze-dried and stored in a −20 °C freezer for further analysis. After washed out by deionized water in the ultrasonication bath for another 30 min, the dissociated lignocellulosic carriers were collected and oven dried at 105 °C for 24 h. Then, the ultimate analysis of grinded lignocellulosic samples before and after utilization as carriers was conducted by an elemental analyzer (Vario Micro cube, Elementar, Germany). Algal biomass samples used for chemical composition analysis were grinded and sieved to gain a particle size below 74 μm. The total lipid content was determined, following the method by Atta et al. [18]. The crude proteins content was estimated by measuring the total Kjeldahl nitrogen following the ASTM E778 and multiplying by the conversion factor of 6.25 [3]. The ash content was quantified according to Gross et al. [3]. The carbohydrate content was achieved by subtracting the total lipid, crude protein, and ash contents from the total weight of the freeze-dried sample.

### Surface characterization of the substrata

Surface physical properties of the materials were determined as dry basis by a confocal laser-scanning microscopy (CLSM) (VK-X100 K/X200 K, KEYENCE, Japan). Three-dimensional (3-D) images of the material surface morphology were taken by a charge-coupled device camera with standard lens (CF IC EPI Plan 20X, Nikon, Japan) under the measurement mode with a scanning area of 705.1 × 500.0 μm^2^. Each type of material was tested eight times with eight randomly selected samples. The images were performed by the software VK-analyzer (VK-H1XAC, KEYENCE, Japan). Wenzel’s roughness factor [19] $r$ was tested through the obtained pictures and calculated, according to Eq. (9):

9 $$r = \frac{1}{8}\sum\limits_{i = 1}^{8} {{\raise0.7ex\hbox{${A_{i} }$} \!\mathord{\left/ {\vphantom {{A_{i} } {A_{0} }}}\right.\kern-0pt} \!\lower0.7ex\hbox{${A_{0} }$}}}$$

where $r$ is the Wenzel’s roughness factor; $A_{i}$ is the tested material surface area of sample $i$; and $A_{0}$ is the geometrically projected area of sample $i$ and equal to 705.1 × 500.0 μm^2^. Afterwards, the images were smoothed and tilt corrected, and then the 3-D surface roughness was determined with the whole scanning area following the ISO 25178. The average surface roughness was defined as Eq. (10):

10 $$S_{\rm a} = \frac{1}{8}\sum\limits_{i = 1}^{8} {S_{{\rm a}i} }$$

$S_{{\rm a}i}$ is the 3-D surface roughness of the sample $i$, while $S_{\rm a}$ is the average 3-D surface roughness of the tested material. As RH has two completely different surfaces, the outer roughness surface and the inner smoothness surface, roughness factor, and average surface roughness of this material are the average value of the two surfaces.

Furthermore, images were applied to test the line roughness of the materials following the ISO 4287-1997 with at least 20 cross-sectional curves for each sample. All the curves were vertical to the orientation of the groove and set with a gap of 20 μm between each other. The average groove depth and the average maximum groove depth were obtained via the software VK-analyzer and defined as follows:

11 $$R_{c} = \frac{1}{8}\sum\limits_{i = 1}^{8} {\left( {\frac{1}{N}\sum\limits_{j = 1}^{N} {R_{{c_{i,j} }} } } \right)}$$

12 $$R_{z} = \frac{1}{8}\sum\limits_{i = 1}^{8} {\left( {\frac{1}{N}\sum\limits_{j = 1}^{N} {R_{{z_{i,j} }} } } \right)}$$

$R_{{c_{i,j} }}$ and $R_{{z_{i,j} }}$ are the average height and the maximum height of the $i$ th sample $j$ th cross-sectional curve, respectively, and they are defined as the average groove depth and the maximum groove depth separately for $i$ th sample $j$ th test and $N \ge 20$. $R_{c}$ and $R_{z}$ are the average groove depth and the average maximum groove depth of the tested materials.

Finally, the software VK-viewer (VK-H1XVC, KEYENCE, Japan) was used to determine the width of the groove existed on the material surface. 3-D images were first divided into 8 × 10 parts with grid, and then the distance between two adjacent peaks in each part was measured and defined as groove width. Therefore, the mean, minimum as well as maximum groove width on the surface of the materials were accordingly calculated via Eqs. (13, 14 and 15):

13 $$\overline{D} = \frac{1}{8}\sum\limits_{i = 1}^{8} {\left( {\frac{1}{80}\sum\limits_{j = 1}^{80} {D_{i,j} } } \right)}$$

14 $$D_{{\rm min} } = \frac{1}{8}\sum\limits_{i = 1}^{8} {\left( {{\rm min} D_{i,j} } \right)} ,\quad j \in \left[ {1,80} \right]$$

15 $$D_{{\rm max} } = \frac{1}{8}\sum\limits_{i = 1}^{8} {\left( {{\rm max} D_{i,j} } \right)} ,\quad j \in \left[ {1,80} \right]$$

$D_{i,j}$ is the tested groove width of the $i$ th sample $j$ th part. $\overline{D}$, $D_{{\rm min} }$ and $D_{{\rm max} }$ represent the mean, minimum as well as maximum groove width of the materials. For RH, the outer roughness surface was used to test $R_{c}$, $R_{z}$, $\overline{D}$, $D_{{\rm min} }$ and $D_{{\rm max} }$.

### Statistical analysis

Experiments were conducted in duplicate or triplicate, and average values were reported. Results were performed with EXCEL 2013 (Microsoft Office Enterprise, USA) and OriginPro 8.0 (OriginLab, USA). One way ANOVA analysis was carried out wherever applicable.

## Results and discussions

### Biomass production

After inoculation for 3 days, a thin layer of bottle green biofilm occurred, proliferated inside the culture channels and covered all of the lignocellulosic materials particles. Algal cells bonded tightly with the carriers and biofilm blends could be harvested very easily as shown in Additional file 1: Figure S1.

Algal biofilm productivity with different materials as substrata is shown in Fig. 2a and b. The small standard deviations demonstrated a good stability and reproducibility of this FPBR system. Different materials as carriers generated significantly different biomass production (*P* < 0.05). After 16 or 20 days’ cultivation, the biofilm production and productivity ranged from 117.21 to 175.96 g m^−2^ and from 7.32 to 10.92 g m^−2^ day^−1^, respectively. In particular, PW showed the greatest productivity of 10.92 g m^−2^ day^−1^ after 16 days’ culturing, followed by SB, OW, and RH in the order of 9.54 > 8.07 > 7.32 g m^−2^ day^−1^. In addition, the production for SB as carrier increased from 152.68 to 175.96 g m^−2^ from 16 to 20 days, while the productivity decreased with the increase of culturing time. Such decrease is consistent with the findings of other reports [3, 14]. According to Fig. 2a and b, all the lignocellulosic materials had better biomass production performances compared to PMMA which had the productivity of only 4.01 g m^−2^ day^−1^. Besides, for the suspended culture, the biomass production and productivity of 2.15 g L^−1^ and 0.13 g L^−1^ day^−1^ were just, respectively, obtained after 16 days under same culture conditions with the biofilm.

To evaluate the potential of a unit weight of materials for algal biomass production, the algal biomass production by g kg^−1^ and productivity by g kg^−1^ day^−1^ were calculated, and are shown in Fig. 2c and d, respectively. The variation trends of the production by g kg^−1^ and productivity by g kg^−1^ day^−1^ were similar to those by g m^−2^ and g m^−2^ day^−1^. However, the productivity difference between the four lignocellulosic materials was enlarged by their bulk density. OW and RH with higher bulk density showed lower productivity when compared with PW and SB with lower bulk density. Therefore, for this new technology, biomass productivity depicted by g kg^−1^ day^−1^ might be an important factor that could give a direct guideline for the selection of the excellent material which could generate more algal biomass comparing with other carriers with the same amount used. According to the results of this study, PW was found to be the best carrier to support the algal biofilm growth with a productivity of 47.52 g kg^−1^ day^−1^ (10.92 g m^−2^ day^−1^) corresponding to a production of 760.37 g kg^−1^ (174.71 g m^−2^) after a period of 16 days culture. The performances of SB (40.16 g kg^−1^ day^−1^), OW (20.63 g kg^−1^ day^−1^), and RH (20.47 g kg^−1^ day^−1^) followed that of PW.

Various types of materials had been used as carriers for the algal biofilm cultivation by researchers. Christenson and Sims [15] tested eight substrata for the ability to support algal attachment and found that cotton rope showed the best performance. While Gross et al. [3] examined more than 16 materials for their capability of supporting algal growth, and cotton duct was proved to offer the highest biomass productivity. Filter paper can also be used as support substrata for algal growth vertically [20]. It is worth noting that these materials were all lignocellulosic-based materials and similar to the tested lignocellulosic materials in this study. They were generally characterized by high surface energy and achieved greater attachment than synthetic polymers which just had low surface energy [15]. Moreover, lignocellulosic materials mainly composed of cellulose, hemicellulose, and lignose were hydrophilic natural polymers and had good liquid-holding capacity [21]. As shown in Additional file 1: Table S1, all the four types of the tested lignocellulosic materials had saturated moisture content larger than 82%, with PW and SB reaching over 90%. Meanwhile, the liquid-holding capacity of the four materials varied similarly to the biofilm productivity. PW (12.49 g/g) and SB (11.49 g/g) had good liquid-holding capacity compared to OW (4.72 g/g) and RH (7.04 g/g). The hydrophilic material with good liquid-holding capacity can be preferable for the attachment cultivation [22]. Many algal species such as *Chlorella*, *Chroococcus*, *Chlorosarcinopsis*, *Synechococcus,* and *Scenedesmus* could be immobilized by hydrophilic polymers [23]. Additionally, few filamentous microorganisms had occurred onside the surface of algal biofilm (not for PMMA as carrier) (Fig. 1a; Additional file 1: Figure S2). Unsterilization of the materials before utilized as carriers probably led to the occurrence of those microorganisms. As a kind of lignocellulosic material, the tested carrier could be degraded by a wide range of microorganism including bacteria and fungi with anaerobic and aerobic habit [24]. However, the positively charged hyphae that fungi had could interact with the negatively charged algal cell surface and cause flocculation [7]. Therefore, co-culture with filamentous fungi could form co-pellets where algae cells were entrapped inside the fungal mycelia and attached on the fungal cell surface [4], as it can be seen from Additional file 1: Figure S3. In addition, co-culture with filamentous fungi could also enhance the algal biomass and lipid production [7]. So the occurrence of filamentous microorganisms could be considered as a beneficial factor for algal biofilm cultivation. These microorganisms might promote the algal cell attachment by retaining the cells onside the substrate layer. Meanwhile, algal productivity could be enhanced by respiration of the microorganism which could provide CO_2_ and decrease O_2_ inhibition for algal growth [25]. Moreover, these microorganisms also produced large amount of extracellular polymeric substances (as shown in Fig. 5), which could bind cells together, solid materials, and form a microenvironment matrix to protect cells from environmental stress [2]. Thus, one or several above reasons led to the better biomass production performance of lignocellulosic carriers compared to the PMMA, which is a hydrophobic material with low surface energy and poor liquid-holding capacity. In addition, many microorganisms like fungi species, similar to algal cells, had high content of lipid [4]. The composition of the microorganisms could contribute to total lipid production of the biofilm, and facilitate the oil extraction of the algal biomass by partially breaking down the algal cell walls [4].

Table 1 shows the ultimate analysis results of the tested lignocellulosic materials before and after algal biofilm cultivation process. As it can be seen from Table 1, no significant difference of element contents existed between the samples before and after utilized as biofilm carriers. Besides, only 1–6.5% (Additional file 1: Table S1) of material mass loss was found for different tested lignocellulosic carriers after 16 or 20 days’ biofilm cultivation process. This indicated that the tested lignocellulosic materials only acted as carriers for algal biofilm development and thus was durable during the biofilm cultivation process. Slight increase of C, H, and N and decrease of O could mainly attribute to the degradation effect of the occurred fungi and the solubilization of light organic compounds from the materials.

**Table 1:** Ultimate analysis of the tested lignocellulosic materials before and after algal biofilm cultivation process (mean ± SD)

Materials	C^a^ (wt%)	H^a^ (wt%)	O^a^ (wt%)	N^a^ (wt%)	S^a^ (wt%)
PW	47.94 ± 0.01	6.24 ± 0.04	45.56	0.15 ± 0.02	0.12 ± 0.02
PW-16	48.63 ± 0.05	6.35 ± 0.01	44.72	0.21 ± 0.01	0.09 ± 0.01
OW	50.10 ± 0.05	6.61 ± 0.05	42.38	0.24 ± 0.01	0.68 ± 0.02
OW-16	50.62 ± 0.03	6.72 ± 0.01	41.59	0.45 ± 0.03	0.62 ± 0.01
SB	44.34 ± 0.11	5.97 ± 0.01	49.18	0.43 ± 0.01	0.09 ± 0.01
SB-16	44.55 ± 0.01	6.09 ± 0.01	48.33	0.95 ± 0.01	0.09 ± 0.01
SB-20	44.50 ± 0.04	6.12 ± 0.01	48.08	1.22 ± 0.02	0.08 ± 0.02
RH	55.19 ± 0.05	6.40 ± 0.04	38.40	0.01 ± 0.00	0
RH-16	55.69 ± 0.03	6.65 ± 0.03	37.62	0.03 ± 0.00	0.01 ± 0.00

XX-*t*, sample of lignocellulosic material; XX, harvested after *t* days’ algal biofilm cultivation process

^a^Dry ash-free basis

### Total lipid content and productivity

Figure 3 shows the chemical contents of the algal biomass. The crude proteins contents of the harvested biomass (all excess 30%) were found to be significantly higher than contents of the total lipid and carbohydrate (*P* < 0.05). High crude proteins content indicated that the harvested biomass could be a suitable feed for animals and aquaculture. However, the total lipid concentrations of biofilm with different lignocellulosic materials as carriers did not vary remarkably (20.10–23.20%), and they were lower than those of PMMA (24.52%) and the suspended culture (28.87%). Their carbohydrate contents were higher than that of PMMA except for PW. Compared to the biofilm, the suspended culture had the maximum crude proteins content (40.07%) and the minimum carbohydrate content (14.85%). Biofilm with lignocellulosic materials as carriers had formed thicker biofilm (see Fig. 1d), which resulted from the already existed thickness of the carriers layer and larger biomass production, leading more cells in the dim light or dark and with lower lipid content. Higher light intensity generally promotes lipid accumulation [26]. Furthermore, the composition of the indigenous microorganisms (see Figs. 1a, 6) inside the biofilm might also contribute to this lower lipid content because of the lower lipid content of indigenous microorganisms compared to the algal cells [8, 27].

Figure 2 illustrates that the total lipid production of the biofilm with different carriers ranged from 15.74 to 40.53 g m^−2^ in the following relationship: PW (40.53) > SB (34.62) > OW (28.42) > RH (23.56) > PMMA (15.74) after 16 days’ cultivation. This indicated that carriers’ difference significantly affected the total lipid production of the biofilm (*P* < 0.05). Moreover, as the culture time increased from 16 to 20 days, the total lipid production increased, reaching to 37.18 g m^−2^ for SB. The production and productivity of crude proteins and carbohydrate were also calculated and are shown in Fig. 4. Combining Fig. 2 with Fig. 4, it was not difficult to find that the highest total lipid and crude proteins productivities occurred in the treatment with PW as carriers, reaching 2.53 and 4.01 g m^−2^ day^−1^, respectively. Meanwhile, SB as carrier induced the greatest carbohydrate productivity of 2.28 g m^−2^ day^−1^, while the lowest yields of chemicals appeared in the treatment using PMMA as carriers, with total lipid productivity of 0.98 g m^−2^ day^−1^, crude proteins productivity of 1.41 g m^−2^ day^−1^, and carbohydrate productivity of 0.88 g m^−2^ day^−1^, respectively. Apart from that, crude proteins productivities of 2.53, 2.78, and 3.01 g m^−2^ day^−1^ were achieved using RH, OW, and SB as biofilm carriers, respectively. The productivities of biofilm chemicals among all carriers were significantly (*P* < 0.05) different, accordingly to statistical analysis. Such differences mainly resulted from the difference in the corresponding biomass productivities, since the deviations in the contents of total lipid, crude proteins and carbohydrate within different treatments were not large in contrast. The crude proteins and total lipid contents of biofilm with PW as carrier were high, and its biomass productivity was also the maximum, resulting in relatively high crude proteins and total lipid productivities. In addition, chemicals productions and productivities calculated as g kg^−1^ and g kg^−1^ day^−1^ varied similarly to those of g m^−2^ and g m^−2^ day^−1^, as depicted in Figs. 2 and 4, and differences among different carriers were enlarged by the corresponding biomass production (g kg^−1^) and productivity (g kg^−1^ day^−1^). Moreover, for the suspended culture, the biomass total lipid, crude proteins, and carbohydrate productivity of only 0.04, 0.05, and 0.02 g L^−1^ day^−1^ was just obtained, respectively, after 16 days under the same culture conditions with the biofilm.

### Effect of surface property on biomass productivity

**Fig. 7:**
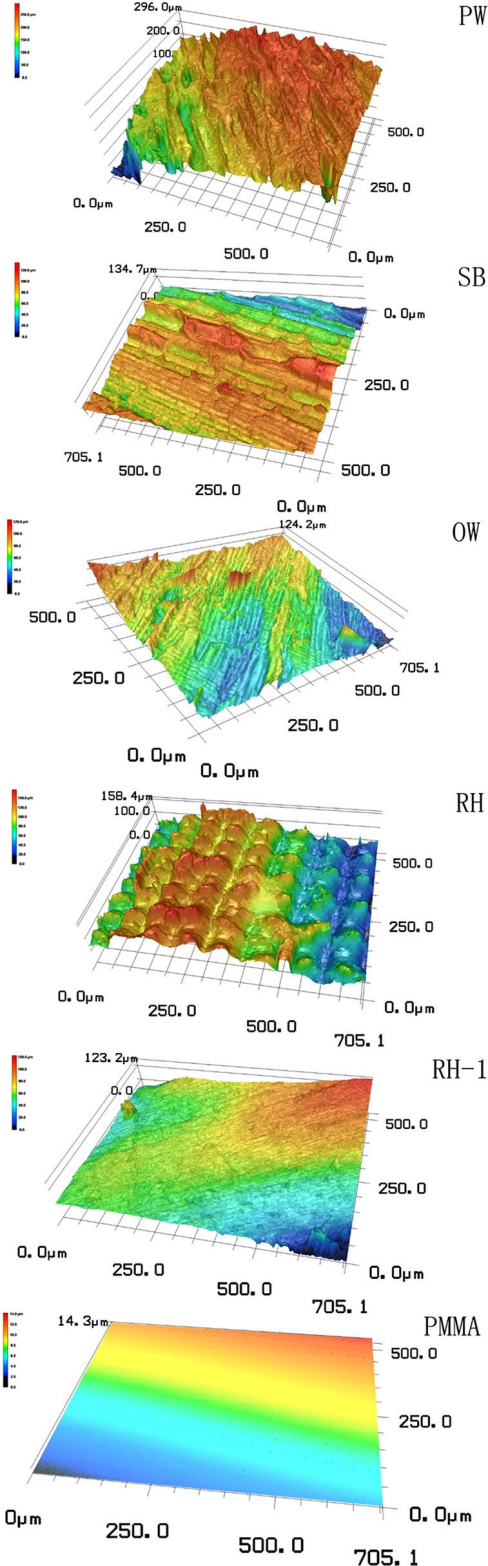
3-D images of the surface topography of the tested five different materials obtained through CLSM technology, RH has two kinds of surfaces with completely different roughness, (RH) the rough outer surface, (RH-1) the smooth inner surface

Confocal laser-scanning microscopy technology was used to determine the surface morphology of the lignocellulosic materials (Figs. 5, 7). As shown in Figs. 5 and 7, surface properties varied significantly among different carriers (*P* < 0.05). For instance, Fig. 5a illustrates a positive correlation (*R* ^2^ = 0.96) between algal biofilm productivity and carriers surface roughness. In addition, the biofilm productivity was found to be increased with the increase of materials surface roughness (*P* < 0.05). Similar trend commonly existed in many reports [16, 28]. Figure 5b demonstrates a reasonable correlation between the material Wenzel’s roughness factor and algal biofilm productivity (*R* ^2^ = 0.96). Materials with larger roughness factor tended to have larger biomass productivity (*P* < 0.05). This indicated that material with relatively bigger surface area would achieve the higher biomass productivity. Cao et al. [29] confirmed that corrosion-resistant steel sheets with micro-dimple surface features could significantly enhance the attachment of algal cells to the substrata, compared to a surface without micro-dimple features. Furthermore, positive correlations between attached pathogenic bacteria (*E. coli*, *Legionella pneumophila*) number and material surface roughness had also been reported in many studies [30, 31]. ESEM images (Fig. 6) and CLSM figures (Fig. 7) demonstrate that all tested lignocellulosic materials had rough surfaces, and were abundant in regular and well-distributed grooves and ridges. However, PMMA surface was much smoother than the tested lignocellulosic materials. The observed grooves and ridges could be formed during particle formation processes by the function of mechanical energy from crashers, pressers, and saws. Among the tested materials, PW (surface roughness 18.98 μm) had the most complicate surface topography with various grooves, similar to the manmade V-groove patterns [19, 32]. Then, the OW and SB had surface roughness of 11.29 and 11.25 μm, respectively. RH had a mean surface roughness of 10.01 μm, the least value among the four materials, but was still larger than that of PMMA (surface roughness of 0.07 μm).

Furthermore, cell attachment had a strong relationship with the size of the grooves [19]. Table 2 lists the message of the grooves on the surface of the substrata. RH had the widest mean size of the grooves, reaching 47.85 μm (the rough surface), followed by SB (26.00 μm), PW (20.44 μm), and OW (15.48 μm). However, PW (49.33 μm) and OW (39.31 μm) had deeper grooves than RH (29.29 μm) and SB (16.68 μm). These features could also be observed in the 3-D images shown in Fig. 7. To a certain extent, the change of groove depth of the tested lignocellulosic materials had the similar trend to biofilm productivity, surface roughness and Wenzel’s roughness factor. Material with rougher surface tends to have a relatively larger surface area, deeper grooves, and a bigger biofilm productivity. Taking these factors into consideration, conclusion can be drawn that surface roughness of material significantly affects biomass productivity mainly through the size of surface morphology and the area of surface.

**Table 2:** The measurements of the grooves on the surface of the tested lignocellulosic materials (mean ± SD)

Material	Groove width (μm)			Groove depth (μm)	
	Mean	Max	Min	Mean	Max
PW	20.44 ± 5.22	31.36 ± 8.25	12.12 ± 9.43	49.33 ± 12.55	82.95 ± 11.38
OW	15.48 ± 2.99	20.65 ± 1.28	12.78 ± 1.33	39.31 ± 7.76	45.47 ± 7.85
SB	26.00 ± 12.83	65.22 ± 24.23	10.88 ± 4.26	16.68 ± 2.35	33.63 ± 6.42
RH	47.85 ± 14.35	79.71 ± 12.35	28.11 ± 8.88	29.29 ± 7.05	46.46 ± 10.79

From the ESEM images (Fig. 6), after 1-day inoculation period and 1-day cultivation, algal cells of *Scenedesmus* sp., *Chlorella* sp., or *Oscillatoria tenuis* preferred to inhabit in the grooves. For PMMA as carrier, however, only few cells existed. Sathananthan et al. [32] patterned the substrata surface with 20 μm V-groove patterns and got an algal productivity double of that smooth surface through 10 days’ mixed culture. Surface micro-topographies which are slightly larger than the size of algal cells would promote attachment, and deeper grooves always achieve more cells attached [19]. In this study, the size of all tested algal species was slightly smaller than the mean depth and the mean width of the observed grooves. Since equal amount of algal inoculum was evenly distributed onside the substrate layers during the seeding process, cells preferred to inhabit into the grooves. Algal cells can be protected from the fluid shear stress through inhibiting into the grooves during the early times. Meanwhile, the formation of the cell clusters and then early biofilm were also protected and promoted by the grooves. But for PMMA with smoothness surface, algal cells inoculated onside it were more easily washed away due to lack of protection that lead to less algal inoculum left. Furthermore, surface with deeper grooves might have recruited more algal cells on the material surface during the seeding process. Therefore, larger amount of inoculum was guaranteed and shorter colonization time was needed [27], and then higher biomass productivity was obtained [19]. Additionally, larger surface for algal cell attachment was provided due to the relatively larger surface area. And materials with rougher surface tended to have higher biofilm adhesion strength, which will then strengthen the resistibility of shear stress [2]. These reasons might together contribute to the fluctuation of the algal biomass productivity with different materials as carriers.

### Comparison with other technologies

Both the new technology and a variety of biofilm technologies are listed in Table 3 with their algal productivities, lipid contents, and culture conditions. It should be noted that the calculation of biomass productivity (g m^−2^ day^−1^) is largely relied on the based surface area, the biofilm growth surface area (*S*_g_), or the footprints area of the reactors (*S*_f_). Higher ratio of *S*_g_/*S*_f_ will tend to generate significantly higher biomass productivities that are calculated based on *S*_f_, if the biomass productivities of different biofilm reactors calculated based on *S*_g_ are the same. Lower biomass productivity with PW as carriers in this study which calculated based on *S*_f_ was mainly due to the lower ratio of *S*_g_/*S*_f_ compared to other technologies. *S*_g_/*S*_f_ of this study was close to 1, which is much smaller than those from Gross et al. [3], Christenson and Sims [15], Liu et al. [14], and Schultze et al. [33]. Amazingly, biomass productivity calculated based on *S*_g_ with PW as carriers was higher than most of the technologies listed above. High contents of total lipid and crude proteins with lignocellulosic materials as carriers were also achieved, and produced biomass could be suitable for energy conversion and animal feed. Most importantly, the high biomass productivities achieved by all the above researchers were coupled with varieties of high-quality materials which were expensive, undurable, or difficult to be obtained, unsuitable for the commercialization of algal biofilm technology.

**Table 3:** Comparison of biomass productivity and total lipid content with different materials as algal biofilm carriers

Materials	Footprint productivity^a^ (g m^−2^ day^−1^)	Surface productivity^b^ (g m^−2^ day^−1^)	Total lipid (%)	Area ratio^c^	Algal species	Conditions: scale, mode, duration day, medium, temperature °C, light intensity μmol m^−2^ s^−1^, CO_2_ (v/v)	References
Pine sawdust	10.92	10.92	23.2	1.04	*Scenedesmus* sp, *chlorella* sp, *oscillatoria tenuis*	Lab, 16 days, initial growth, synthetic medium, 25, 120, 2%	This tudy
Cotton duct	6.84–12.76	1.99–4.99	–	2–4	*Chlorella vulgaris*	Pilot, regrowth, synthetic medium, greenhouse (USA)	[3]
Cotton duct	–	3.51	7.72	2–5	*Chlorella vulgaris*	Lab, 7 days, regrowth, synthetic medium, 25, 110–120	[3]
Membrane (pore size of 5 μm)	13.56	13.56	–	1	*Chlorella* sp.	Lab, 2 days, synthetic medium, 35, 100, 7.5%	[6]
Glass	2.8	2.8	15	1	*Nitzschia palea*	Lab, 14 days, growth, synthetic medium, 26, 160, 16/8, 2%	[8]
Concrete	0.71	0.71	26.8	1	*Botryococcus braunii*	Lad, 35 days, synthetic medium, 25, 55	[12]
Work nylon filter sheets	6.3	–	–	6	*Halochlorella rubescens*	Lad, 54 days, growth, wastewater, greenhouse (Germany)	[13]
Cellulose acetate/nitrate membrane (pore size 0.45 um)	70.9	5.2	47.9	10	*Scenedesmus obliquus*	Lad, 9 days, growth, synthetic medium, 30, 2%	[14]
Cotton rope	31	–	–	2–4	*Mixed culture*	Pilot, 12 days, regrowth, wastewater, outdoor (USA)	[15]
Cotton rope	20	–	11.2	2–4	*Mixed culture*	lab, 20 days, regrowth, wastewater, outdoor (USA)	[15]
Stainless steel woven mesh (particle pass size of 47 um)	–	20.1	–	–	*Chlorella Sorokiniana*	Lab, 7 days, regrowth, synthetic medium, 38, 422, 0.5%	[16]
Plain printing paper	6.1	1.02	–	5.11	*Phaeodactylum tricornutum*	Lab, 16 days, regrowth, synthetic medium, 25, 40–100, 14/10	[20]
Electrostatic flocking cloth	60	–	–	15	*S. platensis*	Lab, 9 days, synthetic medium, outdoor (China)	[22]
Printing paper	50	3	–	15	*Halochlorella rubescens*	Lab, 42 days, synthetic medium, 22, 52	[33]
Polycarbonate membrane	31.2	–	–	–	*Halochlorella rubescens*	Lab, 3 days, synthetic medium, 25, 1023, 3%	[33]
Polystyrene foam	2.57	2.57	9	1	*Chlorella* sp.	Lab, 10 days, wastewater, 20, 110–120	[34]

^a^Algal biomass productivity calculated as dry weight per area of land used by the reactor per unit of time

^b^Algal biomass productivity calculated as dry weight per area of growth surface per unit of time

^c^Area ratio of growth surface area to footprints area of the reactors which calculated based on the parameters of the reactors from the corresponding studies listed above, due to lack of detailed information of some studies, area ratio was given as a conceivable range

Taking this issue into account, this study explored the feasibility of using lignocellulosic materials as biofilm carriers, which had similar chemical composition to algal cell wall. In addition, PW as carriers achieved high biomass productivity (10.92 g m^−2^ day^−1^). Using this new technology for algal biofilm cultivation, seasonal change of the production of the material can be well handled by just in turning utilization of the PW, OW, SB, RH, and other lignocellulosic materials which have not been studied yet in this study. Hence, this new technology with lignocellulosic materials as biofilm carriers has a great potential for the production of algal biomass.

## Conclusions

Using lignocellulosic materials as carriers for algal biofilm growth achieved a productivity ranging from 7.32 to 10.92 g m^−2^ day^−1^, with PW being the maximum value. Total lipid contents of the biofilm with different materials as carriers were less fluctuated (20.10–23.20%), while crude proteins content and carbohydrate content varied significantly with the variation of materials. Total lipid productivity increased significantly as the increase of productivity, and PW gained a maximum value of 2.53 g m^−2^ day^−1^, followed by SB (2.16 g m^−2^ day^−1^), OW (1.78 g m^−2^ day^−1^), and RH (1.47 g m^−2^ day^−1^). Additionally, biomass productivity had a linear relationship with both the surface roughness (*R* ^2^ = 0.96) and roughness factor (*R* ^2^ = 0.96) of the materials. Future work is needed to improve the performance of this technology through optimizing culture conditions and development of newly efficient algal biofilm reactors with high growth surface area-to-footprints area ratio.

## Additional file

**Additional file 1: Table S1.** Particle size, bulk density, liquid holding capacity and mass loss rate ($\alpha$) of the tested lignocellulosic materials. **Table S2.** Settings of the experiments performed inside the flat plate algal biofilm photo-bioreactor with material species, dosage, culture time and number of the involved channel. **Figure S1.** Biofilm using sugarcane bagasse as carrier after 16 days’ cultivation. **Figure S2.** Biofilm with filamentous microorganisms using pine sawdust as carrier after 16 days’ cultivation. Red arrow points to the visible filamentous microorganisms. **Figure S3.** ESEM images of the visible filamentous microorganisms described in Figure S2.

## Abbreviations

BG 11 medium: synthetic medium for blue green algae growth
OD_685_: optical density at wavelength of 685 nm
PW: pine sawdust
RH: rice husk
OW: oak sawdust
SB: sugarcane bagasse
FPBR: flat plate algal biofilm photo-bioreactor
PMMA: poly methyl methacrylate
ESEM: environmental scanning electron microscopy
$W_{\rm b}$: the weight of the harvested and oven dried biofilm blend
$W_{\rm t}$: the weight of the tested carriers before cultivation
$\alpha$: the material mass loss rate (1–6.5%)
$t$: the culture period
$W_{\rm s}$: the weight of the harvested and freeze-dried suspended algal
$r$: Wenzel’s roughness factor
$A_{i}$: the tested material surface area of sample $i$
$A_{0}$: the geometrically projected area of sample $i$ and equal to 705.1 × 500.0 μm^2^
CLSM: confocal laser-scanning microscopy
3-D: three-dimensional
$S_{{\rm a}i}$: the 3-D surface roughness of the sample $i$
$S_{\rm a}$: the average 3-D surface roughness of the tested material
$R_{{c_{i,j} }}$: the average groove depth for $i$ th sample $j$ th test
$R_{{z_{i,j} }}$: the maximum groove depth for $i$ th sample $j$ th test
$R_{c}$: the average groove depth of the tested materials
$R_{z}$: the average maximum groove depth of the tested materials
$D_{i,j}$: the tested groove width of the $i$ th sample $j$ th part
$\overline{D}$, $D_{\hbox{min} }$ and $D_{\hbox{max} }$: the meaning, minimum and maximum groove width of the materials, respectively
*S*_g_: the biofilm growth surface area
*S*_f_: the footprints area of the reactors
