# Supplementary material for: Cultivation of algal biofilm using different lignocellulosic materials as carriers
Source: Biotechnol Biofuels. 2017 May 4;10:115. doi: 10.1186/s13068-017-0799-8 (PMC5418734; doi:10.1186/s13068-017-0799-8)
Supplement: Supplementary file 1 — Additional file 1: Table S1. Particle size, bulk density, liquid holding capacity and mass loss rate ($\alpha$α) of the tested lignocellulosic materials. Table S2. Settings of the experiments performed inside the flat plate algal biofilm photo-bioreactor with material species, dosage, culture time and number of the involved channel. Figure S1. Biofilm using sugarcane bagasse as carrier after 16 days’ cultivation. Figure S2. Biofilm with filamentous microorganisms using pine sawdust as carrier after 16 days’ cultivation. Red arrow points to the visible filamentous microorganisms. Figure S3. ESEM images of the visible filamentous microorganisms described in Figure S2. [file 13068_2017_799_MOESM1_ESM.docx]

**Cultivation of algal biofilm using different** **lignocellulosic materials as carriers**

**Qi Zhang^1^, Cuixia Liu^2^, Yubiao Li^3^, Zhigang Yu^4^, Zhihua Chen^5^, Ting Ye^6^, Xun Wang^6^, Zhiquan Hu^6^, Shiming Liu^6^, Bo Xiao^6^ and Shiping Jin^1*^**

^1^School of Energy and Power Engineering, Huazhong University of Science and Technology, Wuhan 430074, China.

^2^School of Energy and Environmental Engineering, Zhongyuan University of Technology, Zhengzhou 450007, China.

^3^School of Resources and Environmental Engineering, Wuhan University of Technology, Wuhan 430070, China.

^4^Department of Civil and Environmental Engineering, The Hong Kong University of Science and Technology, Hong Kong, China.

^5^School of Environment, Henan Normal University, Xinxiang 453007, China.

^6^School of Environmental Science and Engineering, Huazhong University of Science and Technology, Wuhan 430074, China.

**^*^ Corresponding Author:** Shiping Jin; E-mail: jinshiping@hust.edu.cn

Qi Zhang, E-mail:zhangqi09300218@163.com

Cuixia Liu, E-mail: dy0323@126.com

Yubiao Li, E-mail: Yubiao.Li@whut.edu.cn

Zhigang Yu, E-mail: zgyu2013@hnu.edu.cn

Zhihua Chen, E-mail: chenzhihua@htu.edu.cn

Ting Ye, E-mail: [824643068@qq.com](mailto:824643068@qq.com)

Xun Wang, E-mail: xunw@hust.edu.cn

Zhiquan Hu, E-mail: [huzq@hust.edu.cn](mailto:huzq@hust.edu.cn)

Shiming Liu, E-mail: shmliu@hust.edu.cn

Bo Xiao, E-mail: [xiaobo1958@126.com](mailto:xiaobo1958@126.com)

Table S1 Particle size, bulk density, liquid holding capacity and mass loss rate ($\alpha$) of the tested lignocellulosic materials.

| Material | RH | PW | OW | SB |
| --- | --- | --- | --- | --- |
| Particle size^a^ (μm) | 830 – 1700 | 380 – 830 | 380 – 830 | 380 – 830 |
| Bulk density (kg·m^-3^) | 108.01 | 94.73 | 124.53 | 87.66 |
| Saturated moisture content (%) | 87.48 ± 1.03 | 92.53 ± 0.66 | 82.26 ± 2.18 | 91.94 ± 0.66 |
| Liquid holding capacity (g/g) | 7.04 ± 0.66 | 12.49 ± 1.19 | 4.72 ± 0.71 | 11.49 ± 1.03 |
| $\alpha$- 16^b^ (%) | 6.20 ± 0.15 | 5.00 ± 0.57 | 5.00 ± 0.52 | 1.00 ± 0.22 |
| $\alpha$- 20^c^ (%) | – | – | – | 1.03 ± 0.32 |

^a^value is given as a range;

^b^refers to the mass lose rate of the tested lignocellulosic materials after 16 days’ algal biofilm cultivation process;

^c^refers to the mass lose rate of the tested lignocellulosic materials after 20 days’ algal biofilm cultivation process.

Table S2 Settings of the experiments performed inside the flat plate algal biofilm photo-bioreactor with material species, dosage, culture time and number of the involved channel.

| experiment | condition | Number of the marked channel inside the FPBR | | | | |
| --- | --- | --- | --- | --- | --- | --- |
|  |  | 1 | | 2 | 3 | 4 |
| 1 | Materials | OW | | PW | RH | SB |
|  | Dosage (g) | 5.8 ± 0.2 | | 3.5 ± 0.2 | 5.5 ± 0.2 | 3.5 ± 0.2 |
|  | Culture time (day) | 16 | | | | |
| 2 | Materials | SB | | | | |
|  | Dosage (g) | 3.5 ± 0.2 | | | | |
|  | Culture time (day) | 20 | | | | |
| 3 | Materials | PMMA | | | | |
|  | Dosage (g) | 0 | | | | |
|  | Culture time (day) | 16 | | | | |
| 4 | Materials | OW | PW | | RH | SB |
|  | Dosage (g) | 5.8 ± 0.2 | 3.5 ± 0.2 | | 5.5 ± 0.2 | 3.5 ± 0.2 |
|  | Culture time (day) | 1 | | | | |


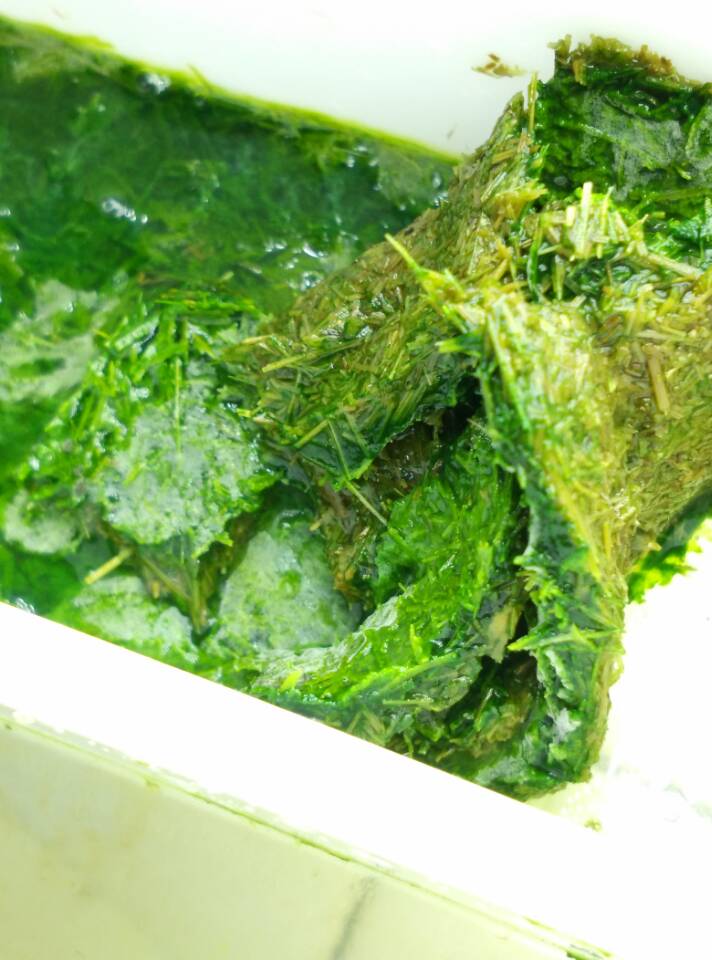


Figure S1. Biofilm using sugarcane bagasse as carrier after 16 days’ cultivation.


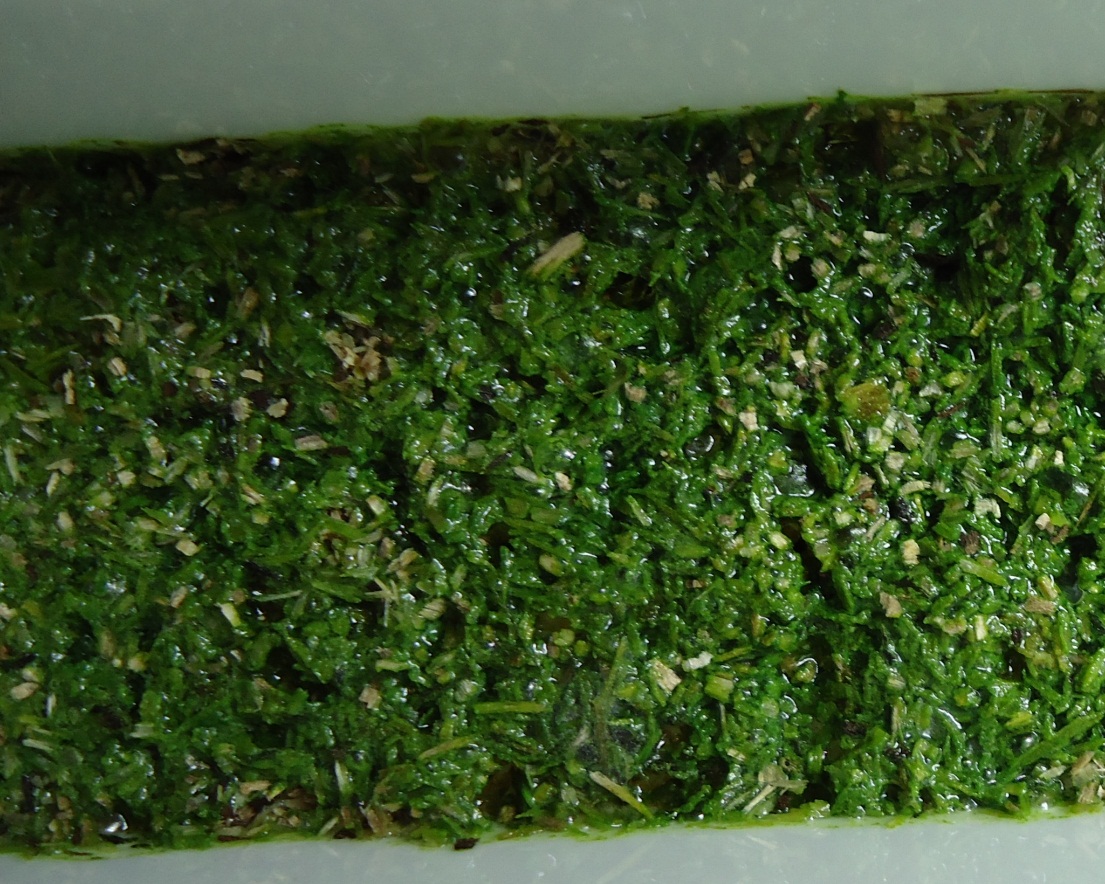


Figure S2. Biofilm with filamentous microorganisms using pine sawdust as carrier after 16 days’ cultivation. Red arrow points to the visible filamentous microorganisms.


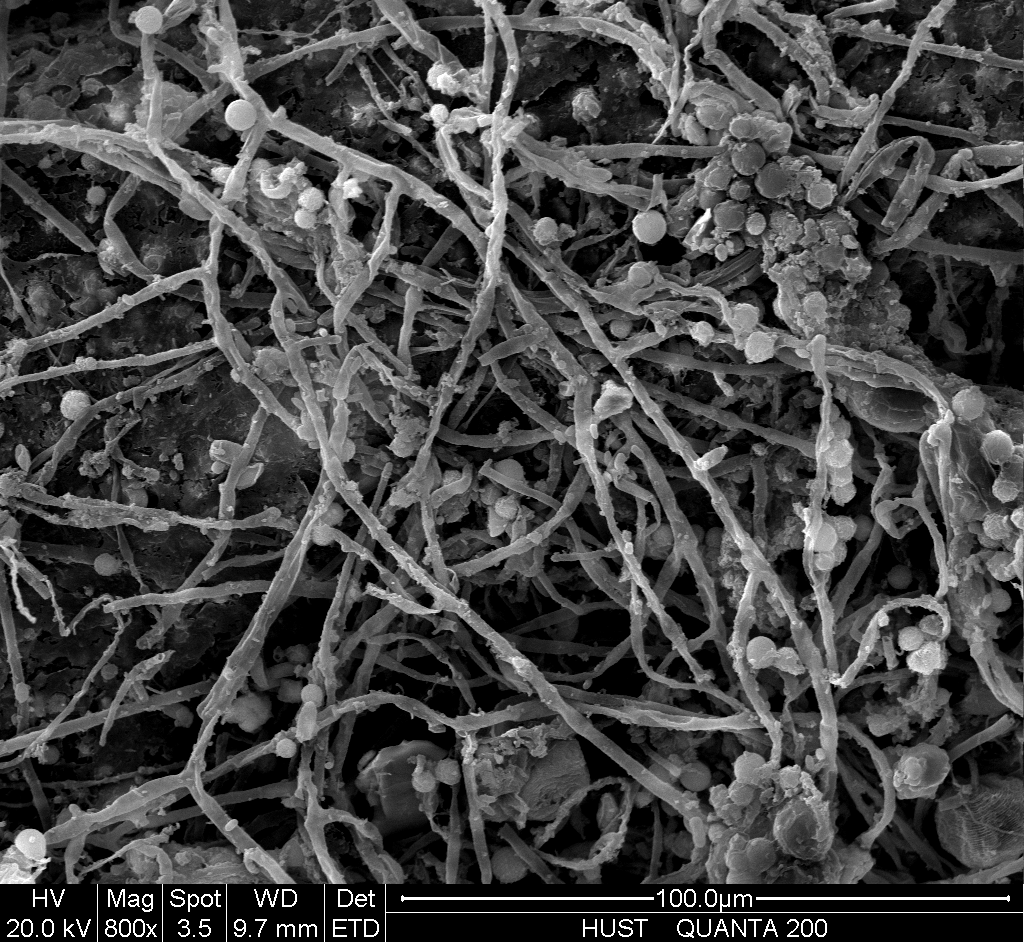


Figure S3. ESEM images of the visible filamentous microorganisms described in Figure S2.
